# A survey of Chinese herbal ingredients with liver protection activities

**DOI:** 10.1186/1749-8546-2-5

**Published:** 2007-05-10

**Authors:** Rubin Wang, John Kong, Dali Wang, Linda Lin-min Lien, Eric Jung-chi Lien

**Affiliations:** 1 Department of Pharmaceutical Sciences, School of Pharmacy, University of Southern California, 1985 Zonal Avenue, Los Angeles, CA 90089-9121, USA; 2 Amylin Pharmaceuticals, Inc, 9360 Towne Centre Drive, San Diego, CA 92121, USA

## Abstract

A literature survey was conducted on herbs, their preparations and ingredients with reported liver protection activities, in which a total of 274 different species and hundreds of active ingredients have been examined. These ingredients can be roughly classified into two categories according to their activities: (1) the main ingredients, such as silybin, osthole, coumarin, glycyrrhizin, saikosaponin A, schisandrin A, flavonoids; and (2) supporting substances, such as sugars, amino acids, resins, tannins and volatile oil. Among them, some active ingredients have hepatoprotective activities (e.g. anti-inflammatory, anticancer, antioxidant, immunomodulating and liver cirrhosis-regulating effects). Calculation of physicochemical parameters indicates that the main ingredients with negative and positive E_lumo _values possibly display their hepatoprotective effects through different mechanisms, such as antioxidative, anti-inflammatory and immunomodulating effects. As the combination of herbs may achieve some treatment effects synergistically and/or additively, it is common in Chinese medicine to use mixtures of various medicinal herbs with pharmacologically active compounds to have synergistic and/or additive effects, or to reduce harmful effects of some pharmacologically active compounds. In particular, the active compounds with Clog P around 2 are suitable for passive transport across membranes and accessible to the target sites. Thus, E_lumo _and Clog P values are good indicators among the calculated parameters.

Seven different physicochemical parameters (MW, Clog P, CMR, μ, E_homo_, E_lumo _and H_f_) and four major biological activities (antioxidant, anti-inflammatory, antiviral/antitumor and immunomodulating) are discussed in this review. It is hoped that the discussion may provide some leads in the development of new hepatoprotective drugs.

## Background

It is well recognized that liver is one of the most important organs in the biotransformation of food, drugs, endogenous and exogenous substances. Profuse supply of blood and the presence of many redox systems (e.g. cytochromes and various enzymes) enable liver to convert these substances into different kinds of inactive, active or even toxic metabolites. The burden of metabolism and exposure to dangerous chemicals make liver vulnerable to a variety of disorders, such as acute or chronic inflammation, toxin-/drug-induced hepatitis, cirrhosis and hepatitis after viral infection.

For centuries, many herbs have been used as natural remedies for the prevention and/or treatment of liver diseases. Various herbs and herbal products are believed to have liver protective functions and widely used in clinical practice in the West as well as East. In the First Edition of PDR for Herbal Medicine [1], for example, a total of 49 herbal products were listed under the preparations for clinical use in liver and gall bladder complaints and 32 products for liver disorders. Some complex Chinese herbal formulae, such as Pro-liver Pill (*Yanggan Wan*)[2], Liver Care (Himalaya Drug Co, Bangalore, India), Liv-52 [3], *Jianpi Wenshen *Pill (*Jianpi Wenshen Wan*) [4], *Binggan *capsules (*Binggan Jiaonang*) [5], *Binggan Tang *[6], *Yizhu *decoction(*Yizhu Koufuye*) [7], *Yiergan Tang *[8] and *Xiaochaihu Tang (Sho-saiko-to *or SST,) [9], have been reported to have significant therapeutic effects on liver protection or treatment of liver diseases. It is yet to be established as to which of the herbs and their active ingredients in these formulae contribute most to the activities of liver protection and treatment.

Both manual and electronic search of literature and/or biological information in herbal medicine was carried out in order for us to examine which structural and physicochemical factors probably affect hepatoprotection. Based on two reference books, namely *Zhongcaoyao Xiandai Yanjiu *[10-12] and *The Chemical Constituents of Oriental Herbal Drugs (Vol 1 & 2) *[62,63], electronic literature search was performed with "ovid"(an online resource with access to multiple databases such as Medline, Biosis, Embase, Current Contents, as well as journals and books), by the key words of "herbal medicine with hepatoprotective activity" and was mainly focused on the literature from 1995 to 2005. In this review, most of the reported ingredients of the heptoprotective herbs are summarized and listed under family, genus and species names of the herbs (Additional file 1) and seven physicochemical parameters of the hepatoprotective compounds, namely molecular weight (MW), calculated octanol/water partition coefficient (Clog P), molar refraction (CMR), dipole moment (μ), energy of the highest occupied molecular orbital (E_homo_)_, _energy of the lowest unoccupied molecular orbital (E_lumo_) and heat of formation (H_f_), have been calculated and reported (Additional file 3). Chemical structures of some of the representative compounds together with their respective calculated physicochemical parameters are also provided (Additional file 2).

## Biological activities of extracts and ingredients of medicinal herbs

According to the results from our literature survey, nearly 300 species of plants belonging to 92 different families have been reported to have liver protection or therapeutic effects on hepatic disorders (Additional file 1). Families of *Compositae, Labiatae and Leguminosae *cover the largest numbers of species.

These plant species usually contain phytochemicals such as phenols, organic acids, flavonoid glycosides, tannins, resins, amino acids, sugars, alkaloids, saponins and volatile oil as well as their own unique constituents. It is not clear whether one, several or all of these components are active ingredients for liver protection. It is likely that some specific ingredients of each herb play a vital role in liver protection/treatment, thereby contributing to their therapeutic effects. Structures of some representative liver protective compounds [10-34] and key physicochemical parameters which may affect their pharmacokinetic and pharmacodynamic effects are provided (Additional file 2).

Among the many hepatoprotective herbs/compounds, milk thistle (*Silybum marianum*) used in the West [35], glycyrrhizin, *Bupleurum chinense *(Saiko), *Schisandra chinensis *(*Wuweizi*) and *Phyllanthus amarus *used in traditional Chinese medicine have been most extensively studied and documented [36].

**Figure 1:**
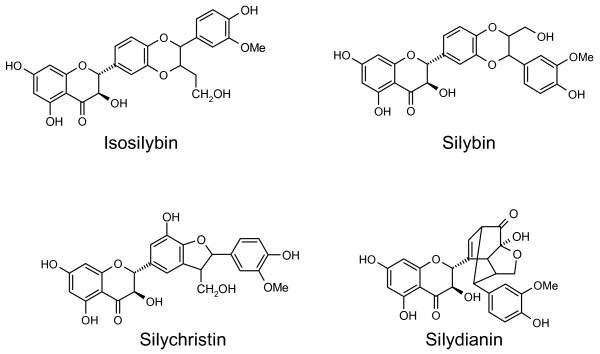
Structures of the four isomers in silymarin.

Silymarin, a mixture of flavonolignans extracted from the seeds of *Silybum marianum*, is most widely used as a remedy for liver diseases. It is composed of four isomers: the major active component silybin, isosilybin, silychristin and silydianin (Figure 1). Moreover, other seed constituents of *Silybum marianum *such as betaine, other flavonolignans and essential fatty acids from the same plant have been shown to have anti-inflammatory properties [37]. The beneficial effects of silymarin are most often observed in patients with cirrhosis as a result of alcohol abuse [38]. But it does not reduce mortality; nor does it improve liver histology or biochemical markers of liver function among patients with chronic liver diseases [35]. It was reported that treatment with *Silybum marianum *and its constituents appears to be safe with no signs of adverse effects [15].

**Figure 2:**
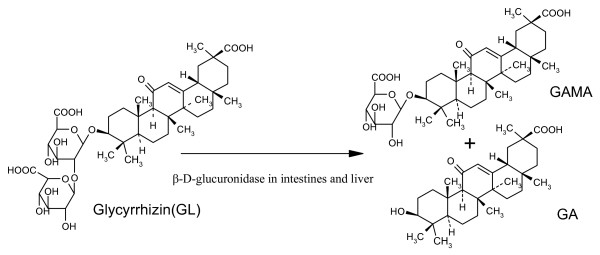
Metabolism of glycyrrhizin catalyzed by β-D-glucuronidase in human liver and intestines.

Glycyrrhizin (GL), isolated from the water-soluble extract of *Glycyrrhiza uralensis*, has been orally or intravenously administered for the treatment of chronic viral hepatitis [34]. It was discovered that GL is metabolized into 18β-glycyrrhetinic acid (GA) and 18β-glycyrrhetinic acid-3-O-β-D-glucuronide (GAMA) by β-D-glucuronidase in the liver and intestines [39,40] (Figure 2).

Structure-bioactivity relationship studies indicate that GA is the most active ingredient against platelet aggregation, tumor cell lines, rotavirus infection and growth of *Helicobacter pylori*, followed by GAMA and GL [41]. Thus, GA is a better hepatoprotective drug than GAMA or GL. It has been suggested that GL (a natural prodrug and sweet principal of Glycyrrhiza) and its hydrolytic metabolites (GAMA, GA) bind to hepatocytes to modify the expression of HBV (hepatitis B virus)-related antigens and suppress sialylation of hepatitis B virus surface antigen (HbsAg) [34,42]. Clinical studies showed that GL has comparable antiviral effects to those of interferon and that it is effective in reducing complications of chronic hepatitis C without apparent side-effects [43,44].

**Figure 3:**
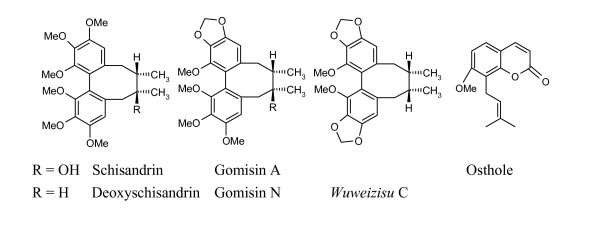
Structures of the dibenzocyclooctadiene compounds and osthole.

*Schisandra chinensis *(*Wuweizi*), traditionally used as a tonic, is now widely used for the treatment of chemical/viral hepatitis. Biological studies indicate that seed extract of *Schisandra chinensis *can enhance hepatic GSH (glutathione) antioxidant/detoxification system and facilitate both processes in the liver. It may be a promising agent for improving phase I oxidative metabolism in liver damaged by CCl_4_[45]. Chemical investigations on the extract have revealed the presence of lignans with a dibenzocyclooctadiene skeleton such as schisandrin, gomisin A, deoxyschisandrin (schisandrin A), γ-schisandrin and *wuweizisu *C [46] (Figure 3). These components are believed to be active in hepatoprotective, anti-inflammatory, anticancer, anti-HIV and immunomodulating effects [47-50].

Structure-activity relationship (SAR) analyses suggest that the presence of methylenedioxy/a hydroxyl group in the dibenzocyclooctadiene skeleton is an important structural feature for hepato- and neuro-protective activities [51,52]. Osthole, a coumarin derivative contained in many medicinal plants especially in *Boenninghausenia albiflora*, was reported to protect against liver injury, and these effects are related to the presence of 7-methoxy and 3-methyl-2-butenyl groups in the structure [53,54]. Due to its low toxicity, osthole appears to be suitable for the treatment of chronic hepatitis and can be used as a lead compound for further development of orally ingested hepatoprotective drugs.

**Figure 4:**
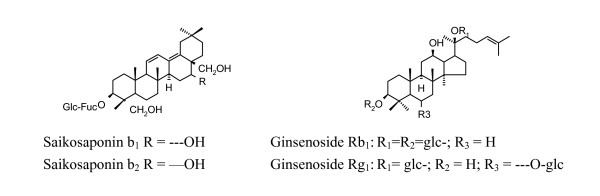
Structures of the saikosaponin and ginsenoside compounds.

Apart from single herbal medicine, 'Kampo medicine' which utilizes combinations of raw herbs, was widely used in Japan, among which SST is the most well-known. It is a spray-/freeze-dried extract from seven medicinal herbs (*Bupleurum falcatum, Glycyrrhiza glabra, Panax ginseng, Pinellia ternate, Scutellaria baicalensis, Zizyphus jujube and Zingiber officinale*). Many clinical and experimental studies demonstrated that SST has a variety of therapeutic effects ranging from anti-inflammatory, antioxidant immunomodulating to hepatoprotective, especially on chronic hepatitis. HPLC (high performance liquid chromatography) analysis of pharmacologically active ingredients in SST indicated that the major fractions of SST contain compounds of flavonoid-like structures (e.g. baicalin, baicalein, liquiritin) and triterpene saponins (GL, saikosaponins b1 & b2, ginsenosides Rg1 and Rb1) [55] (Figure 4). Comparative experiments showed that the liver concentrations of the active constituents of SST extract in the liver-injured rats increased significantly after the animals received SST extract, as compared with the groups receiving the respective purified constituents. Thus, administration of SST extract was more useful than that of its active ingredients individually [56].

In summary, the ingredients as stated above possess hepatoprotective activities, ranging from antiviral, anti-inflammatory, anticancer, antioxidant to immunomodulating and liver cirrhosis regulating effects. Why do the active constituents with different structural features have common biological activities? There may be some innate factors affecting liver protective activities.

## SAR analysis of some hepatoprotective ingredients

A total of 82 representative compounds belonging to nine major groups (i.e. oleanolic acids, hederagenins, polysaccharides, rehmanniosides, xanthine derivatives, alkenes & alkanes, camelliagenin & theasapogenol, campenosides & flavonoids, and aromatic compounds) have been reported to have hepatoprotective effects (Additional file 3). Seven different physicochemical parameters, namely the Clog P, CMR, μ, E_homo_, E_lumo_, H_f _and MW, have been selected as molecular indicators:

(1) E_lumo _values can be divided into three subsets ranging from -1.12 ~ -0.10, and 0.03 ~ 1.07, to 1.10 ~ 2.12 (Additional files 2 and 3). As E_lumo _values reflect electron affinity of the respective compounds [57], these positive and negative ranges may indicate different mechanisms of actions in hepatoprotection. For example, the E_lumo _values of *Silybum marianum*, osthole, coumarin, as well as flavonoids and their glucosides, ranging from -1.03 to -0.83, suggest that their hepatoprotective activities may be achieved via their antioxidative properties, while glycyrrhizin, *Schisandra chinensis *and *Bupleurum chinense*, with their E_lumo _values ranging from 0.03 to 1.07, possibly exert their liver protection through other mechanisms (e.g. anti-inflammatory and/or immunomodulating).

(2) Clog P value, a measure of the hydrophobicity of the compound, may contribute to hydrophobic interactions when the compound binds to the receptor. Different series of compounds vary considerably in their ClogP values (Additional file 3). Polysaccharides, rehmanniosides, rutin, caffeine and theophylline have negative Clog P values ranging from -0.06 to -7.82. Conversely correlated with the number of sugar moieties attached to the corresponding structures, the Clog P values of camelliagenins, theasapogenols, oleanolic acids and hederagenins are mostly above 4. The presence of sugar units decreases the partition coefficient of the core structures in the direction of the values getting closer to the ideal log P_0 _of 2 and facilitates transmembrane absorption in human body before the compounds' binding to the receptors [58-60]. Other series of compounds have Clog P between 0.7 and 4.0. The more active compounds in hepatoprotection have more favorable Clog P values around 2, such as silybin (1.94), glycyrrhizin (1.89) and coumarin derivatives (1.4~3.7), which are suited for passive transport across membranes and make it easier for the compounds to access the target sites (Additional file 2).

(3) An indicator of the potential of dipolar interactions among molecules, μ may have some effects on transmembrane absorption as well as interactions with receptors once the molecules are administered and transported via body fluid. The dipole moment values can be roughly divided into three subgroups: high (9.26 – 6.12), medium (5.98 – 1.62) and low (1.45 – 0.24) (Additional file 3). It was anticipated that compound with high or low value of dipole moment would not possess ideal biological activity because it was difficult to cross multiple biological membranes to reach active sites. Some prodrugs with high values of dipole moment (glycyrrhizin: 7.01) may still display some biological activities after metabolic removal of the polar group (18 *β*-Glycyrrhetinic acid: 5.83). Only the compounds with medium dipole moment values possibly have satisfactory hepatoprotective activities (silybin: 1.92; osthole: 3.77). Finally, MW and H_f _values of these compounds may have some effects on their biological activities in their own ways (Additional file 3).

**Figure 5:**
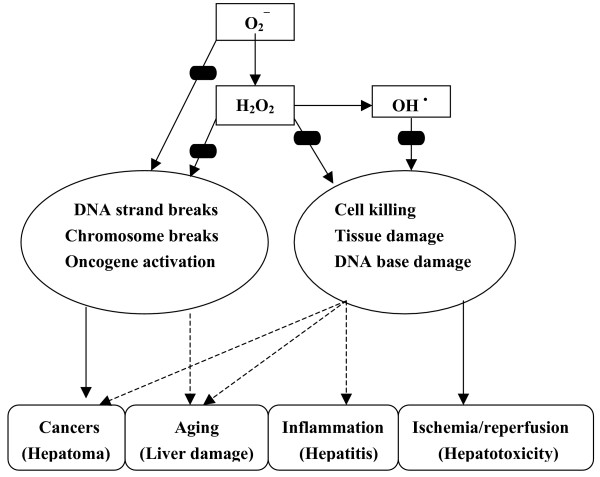
Damaging effects of oxyradical on biological systems and sites of blockades () by antioxidants/free radical scavengers (adapted and expanded from reference [61]).

Although biological effects of these ingredients have not been fully elucidated by these data, the antioxidant activities are indicated in their E_lumo _values. It is speculated that the compounds with negative E_lumo _values may act as free radical scavengers by having one electron from the highest occupied molecule orbital (HOMO) changed to the lowest unoccupied molecule orbital (LUMO), thereby eliminating the damaging effects of free radicals. The damaging effects of oxidation on the biological system via radical mechanism and the hepatoprotective effects by antioxidants and/or free radical scavengers are illustrated in Figure 5[61].

## Conclusion

Herbal medicine has been used in China for more than two thousand years and is now being gradually accepted worldwide, particularly in Asia, Europe and the North America. Differences, however, do exist between the East and West in the areas such as philosophical view point, concept of diseases and treatment approaches. In the West, people tend to use a single herb in therapies and try to identify active ingredients and therapeutic mechanisms, whereas in Chinese medicine it is common to use mixtures of various medicinal herbs which contain a range of pharmacologically active compounds working additively/synergistically to treat patients or to reduce harmful effects of some chemical compounds. It is our hope that the integration of Chinese and Western medicine will not only identify different ingredients with similar bioactivities or similar ingredients with different bioactivities, but also provide us with methods for quality control in herbal medicine and clues to elucidate action mechanisms of active ingredients.

This review is a summary of 274 species (in 216 genera among 92 families) of herbs that have been reported to have positive effects on liver protection and treatment, as well as hundreds of active ingredients. These ingredients can be roughly classified into two categories: (1) the main ingredients, such as silybin, osthole, coumarin, glycyrrhizin, saikosaponin A, schisandrin A, flavonoids, and (2) the supporting substances, such as sugars, amino acids, resins, tannins and volatile oil. Calculations of molecular parameters suggest that negative and positive E_lumo _values in the main ingredients represent two different hepatoprotective mechanisms. The main ingredients with negative E_lumo _values may display their hepatoprotective effects through an antioxidative mechanism, whereas other ingredients with positive E_lumo _values may act via an anti-inflammatory and/or immunomodulating mechanism. Moreover, the Clog P values of many pharmacologically active compounds are around two and other ingredients with higher or lower Clog P values usually go through metabolic processes in which some hydrophilic groups are added or removed so that their Clog P values are around two. E_lumo _and Clog P values, therefore, should be useful in the future development of hepatoprotective drugs.

## Abbreviations

MW:Molecular weight

Clog P: Calculated octanol/water partition coefficient

CMR: Molar refraction

μ: Dipole moment

E_homo_: Energy of the highest occupied molecular orbital

E_lumo_: Lowest unoccupied molecular orbital

H_f_: Heat of formation

SST: *Sho-saiko-to*

GL: Glycyrrhizin

GAMA: 18β-glycyrrhetinic acid-3-O-β-D-glucuronide

HBV: Hepatitis B virus

HbsAg: Hepatitis B virus surface antigen

GSH: Glutathione

SAR: Structure-activity relationship

HPLC: High performance liquid chromatography

HOMO: Highest occupied molecule orbital

LUMO: Lowest unoccupied molecule orbital

## Competing interests

The author(s) declare that they have no competing interests.

## Authors' contributions

RW compiled all data and conducted the computation of the physicochemical parameters used and writing of the manuscript. JK and DW assisted in the literature search and the computation of the parameters used. LLL and EJL contributed equally in the editing of the manuscript and literature survey/interpretation.

## Supplementary Material

Additional File 1 Plants reported to have liver protection activities. The table provides genus and species names of the hepatoprotective herbs, and their reported ingredients. Click here for file

Additional File 2 Representative structures of compounds with potential hepatoprotection and their key physicochemical parameters (MW, Clog P, CMR, μ and E_lumo_). The table presents the chemical structures of the compounds with potential liver protection and the corresponding physicochemical parameters, namely MW, Clog P, CMR, μ and E_lumo_. Click here for file

Additional File 3 The hepatoprotective compounds and the calculated physicochemical parameters used in analysis. The data provided represent the physicochemical properties of the compounds with potential hepatoprotection. Click here for file
