# Supplementary material for: A survey of Chinese herbal ingredients with liver protection activities
Source: Chin Med. 2007 May 10;2:5. doi: 10.1186/1749-8546-2-5 (PMC1876451; doi:10.1186/1749-8546-2-5)
Supplement: Additional File 1 — Plants reported to have liver protection activities. The table provides genus and species names of the hepatoprotective herbs, and their reported ingredients. [file 1749-8546-2-5-S1.doc]

**Plants reported to have liver protection activities**

| *****Family names | *****Latin names | Ingredients**^[62, 63, a]^** | |
| --- | --- | --- | --- |
|  |  |  | |
| Acanthaceae | *Acanthus ilicifolius* | [α-L-Arabinofuranosyl (1→4) β-D-glucuro-pyranosyl (1→3) ]3-β-hydroxyl-up-20(29)ene | |
|  | *Andrographis paniculata* | Andrographolide, Andrographan, Androgra-phon, Deoxyandrographolide, Neoandrograph-olide, Homoandrographolide, Panicolide, Andrographosterin, *β*-Sitosterol-*D*-glucoside, Mono-*o*-methylwightin, Andrographin, Panic-olin,Apigenin-7,4’-dimethylether, *α*_1_-Sitosterol, 14-Deoxy -11-oxoandrographolide, 14-Deoxy-11,12-didehydroandrographolide, 5-Hydroxy-7,8-dimethoxyflavone, Paniculides A, B, C | |
|  | *Rostellularia procumbens* | Justicidin C, D | |
|  |  |  |  |
|  | *Rungia pectinata* |  | |
| Actinidiaceae | *Actinidia chinensis* | Actinidine, Carotenoids, Ascobic acid, Organic acids | |
|  | *Actinidia purpurea* |  | |
| Anacardiaceae | *Cotinus coqqygria* | Gallic acid, Tetrasacchride, Trigalloylglucose, Trimethylgalloylglucoe, Delphinidin, Idaein, Cyanidin-& petunidin-3-galactosides, Cyanidin monoglucosides, Peonidin, Delphinidine | |
| Apocynaceae | *Alstonia yunnanensis* | Sarpagine, Vellosimine | |
|  | *Kopsia officinalis* | Kopsinine, Indole alkaloids, N-Methoxy-carbonyl-11,12-methylenedioxykopsinaline | |
|  | *Wrightia pubescens* |  | |
| Aquifoliaceae | *Ilex rotunda* | Flavonoid glycoside, Phenols, Tannins, Triterpenoid saponin, *β*-Amyrin, *β*-Sitosterol, Oleanolic acid, Rotundic acid, Ilexin A & B, Amino acid, Carbohydrates, Ilex saponin | |
| Araliacea | *Aralia armata* |  | |
|  | *Aralia chinensis* | Triterpenoid saponin, Tannin, Choline | |
|  | *Aralia elata* | Cardiac glycoside, Saponin, Araloside A, B, Oleanolic acid | |
|  | *Hedera nepalensis* | Tannin, Resin, Hederin, Carotenoids, Inositol, Carbohydrates | |
|  | *Cynanchum amplexicaule* |  | |
| Asclepiadaceae | *Cynanchum mamoenum* |  | |
|  | *Marsdenia teracissima* | Cissogenin | |
|  | *Tylophora yunnanensis* |  | |
| Asteraceae | *Cichorium intybus* | Azelaic acid, Daucosterol,Lactucopicrin,8-Desoxylactucin,Crepidiaside B, Sonchuside A, Ixerisoside D, Cichoriin-6’-p-hydroxyphenyl acetate, 11*β*,13-Dihydrolactucin, Jacquinelin, Lactucin, 8-Desoxylactucin, Lactucopicrin*,* Crepidiaside B, Cichorioside B | |
|  | *Bidens bipinnata* | Alkaloids, Tannins | |
|  | *Carpesium abrotanoides* | Capesialactone, Carabrone, *n*-Caproic acid, Oleic acid, Linoleic acid, Granilin, Oleic acid, *n*-Hexanoic acid, (+)Linolenic acid, Stigmasterol, Hentriacontane | |
|  | *Cephalanoplos segetum* | Alkaloids, Saponins | |
|  | *Cichorium intybus* | Esculetin, Esculin, Cichorin, Lactacin, Lactacopicrin, *α*-Lactacerol, Anthocyanin, Monocaffeyltartaric acid, Chicoric acid | |
|  | *Crepis phoenix Dunn.* |  | |
|  | *Dichrocephala benthamii* |  | |
|  | *Elephantopus scaber* | Epifriedelinol, Lupeol, Stigmasterol, Triacontan-1-ol, Dotriacontan-1-ol, Lupeol acetate, Deoxyelephantopin, Isodeoxyeleph-antopin, 11,13-Dihydrodeoxyelephantopin | |
|  | *Erigeron annuus* | Ent-11*α*-hydroxy-15-oxokaur-16-en-19-oic acid, Pyromeconic acid, Quercetin, Apigenin-7-glucuronide | |
| Compositae | *Galinsoga parviflora* | Falcarinone | |
|  | *Inula cappa* | Volatile oil, Flavonoid, Phytosterols, Phenols, 3,5,7,2’-Tetrahydroxyflavanone, *l*-Inositoltetra-angelates, 7,5’-Dimethoxy-3,5,2’-trihydroxy-flavone, (2*S*),5,7,2’,5’-Tetrahydroxyflavanone, (*2R, 3R*)-5’-methoxy- *β*-Farnesene, Squalene, Caryophyllene oxide | |
|  | *Kalimeris indica* |  | |
|  | *Lxeris gracilis* |  | |
|  | *Pentanema indicum* |  | |
|  | *Senecio nemorenis* | Macrophylline, Sarracine, Cynarin | |
|  | *Siegesbeckia orientalis* | Orientalide, Darutigenol, 16,17-Dihydroxy-16- *β*-(-)kauran-19-oic acid, 6,15,16-Trihydroxy-enantiopimar-8 (14)-ene-6- *β*-*D*-glucoside, 16 *β*-(-)-Kauran-17,19-diacid, 16,17-Dihydroxy-16*β*-(-)-Kauran-19-oic acid, Falcarinone | |
|  | *Silybum marianum* | Sugars, Lipids, Silybin, Silydianin, Silychristin, Silandrin, Isosilybin, Silymonin, Silyhermin, Neosilyhermin A & B, Vitamin C, E, K, Alkaloids, Saponins, Essential oil, Fatty acids, Mucilages, Histamine, Tyramine, Betaine, Trimethylglycine, Taxiflin, Dehydrokaemp-ferol, Kaempferol, Kaempferol-7-glucoside-3-sulphate, Apigenin, Apigenin-7-O-glucoside, Apigenin-7-O-glucuronide-4’,7-diglucoside, Luteolin and its glucoside, Quercetin, Acetate triterpene, *β*-Sitosterol and its glucoside | |
|  | *Sonchus brachyotus* |  | |
| Compositae | *Taraxacum mongolicum* | Taraxasterol, Choline, Inulin, Pectin, |  |
|  | *Taraxacum officinale* | Taraxerol, Sitosterol, Taraxol |  |
|  | *Vernonia cinerea* | Flavonoid glycoside, Phenols, Amino acids |  |
| Convolvulaceae | *Ipomoea hungaiensis* |  |  |
| Cornaceae | *Cornus capitata* |  |  |
| Crassulaceae | *Orostachys fimbriatus* | Oxalic acid |  |
|  | *Sedum leucocarpum* |  |  |
|  | *Sedum sarmentosum* | N-Methylisopelletierine, Sedoheptulose, Sucrose, Fructose |  |
| Cruciferae | *Isatis tinctoria* | Indoxyl-*β*-glucoside, *β*-Sitosterol, Isatin, Anthraquinone, Tryptanthrin, 6,12-Dihydro-6,12-dioxoindolo-(2,1b)-quinazoline, Sinigrin, Myrosin, Allylisothiocyanate |  |
| Cucurbitaceae | *Cucumis melo* | Elaterin |  |
|  | *Hemsleya macrosperma* | Saponins(→Oleanolic acid), Dihydrocu-curbitacin F-25-acetate, Bitter principles |  |
| Cyperaceae | *Kyllinga brevifolia* |  |  |
| Dipsacaceae | *Triplostegia grundiflora* |  |  |
| Ebenaceae | *Diospyros rhombifolia* |  |  |
| Elaeagnaceae | *Elaeagnus glabra* |  |  |
| Equisetaceae | *Equisetum debile* |  |  |
|  | *Equisetum ramosissimum* | Kaempferol-3-sophoroside |  |
| Ericaceae | *Agapetes mannii* |  |  |
|  | *Baliospermum effusum* |  |  |
|  | *Euphorbia helioscopa* | Quercetin-5,3-di-*D*-galactoside, Helioscopiol, Phasin, *β*-Dihydrofucosterol, Euphoscopin A and B (toxic substances) |  |
|  | *Euphorbia milii* |  |  |
| Euphorbiaceae | *Homonoia riparia* |  |  |
|  | *Mallotus apelta* | Phenols, Tannin, Amino acid, Carbohydrates |  |
|  | *Phyllanthus amarus* | Lignans such as Phyltetralin, Nirtetralin, Niranthin, Hypophyllanthin, Phyllanthin |  |
|  | *Phyllanthus urinavia* | Tannins |  |
|  | *Sapium sebiferum* | Xanthoxylin, Eriodictyol-5-O-methylether-7-O- *β*-*D*-arabinopyranoside, Flavanone |  |
| Gentianaceae | *Canscora lucidissim* |  |  |
|  | *Centaurium pulchellum* |  |  |
| Gentianaceae | *Gentiana rhodantha* |  | |
|  | *Halenie corniculata* | 1-Hydroxy-2,3,4,7-tetramethoxyxanthone, 1-Hydroxy-2,3,4,5-tetramethoxyxanthone,  1-Hydroxy-2,3,5-trimethoxyxanthone | |
|  | *Swertia erythrosticta* |  | |
|  | *Swertia pulchella* | Flavonoids, Alkaloids, Coumarin | |
|  | *Swertia punica* |  | |
|  | *Swertia vacillans* |  |  |
|  | *Swertia yunnanensis* | Hydroxyl & methoxyl derivatives of anthraquinone, Glycosides of lactone & flavonoid, Anthraquinone glycosides, | |
| Gesneriaceae | *Didymocarpus* | Alkaloids, Palustrine, Palustridine, Aconitic acid, Nicotine (trace) | |
|  | *fimbrispalus* |  | |
|  | *Hordeum dislichon* | Maltoxine (Cundicine) | |
|  | *Imperata cylindrical* | Citric acid, Oxalic acid, Malic acid, Starch, Carbohydrates | |
| Gramineae | *Melica scabrosa* |  | |
|  | *Pogohatherum crinitum* | Flavonoid glycosides, Phenols, Amino acids, Carbohydrates, Organic acids | |
|  | *Zea mays* | Stigmasterol,Vitamin B_5_,C K, Sitosterol, Coixol, Maysin, Zeaxanthin, Cyclosadol, 2”-O-*α*-*L*-Rhamn-osyl-6-C-(6-deoxyxylohexos-4-ulosyl) apigenin & luteolin, 24-Methyl-*E*-23-dehydrol-ophenol & -cholesterol, Fatty & volatile oils, Gum-like substance, Resin, Saponin, Alkaloids, Organic acids, | |
| Hydrocharitaceae | *Ottelia alismoides* |  | |
|  | *Clusiaceae guttiferae* |  | |
|  | *Damna canthal* |  | |
| Hypericaceae | *Hypericum bellum* |  | |
|  | *Hypericum japonicum* | Flavonoids, Lactone, Tannins, Anthraquinones, Phenols, Coumarin, Amino acids | |
|  | *Hypericum patulum* |  | |
|  | *Hypericum perforatum* | Tannin, Volatile oil including:Pinene, Sesqui-terpenes, Resins (containing Hyperesin I, II) Vitamin C, Carotenoids, Rutoside, Hyperoside, Quercitrin, Quercetin, Hypericin Chlorogenic acid, Saponin , *β*-Sitosterol, Capric aldehyde, Lauric acid, Mannitol, | |
| Iridaceae | *Iris dichotoma* | Flavonoid, Isoflavonoid, Tectoridin, Iridin, Pigments | |
|  | *Iris japonica* |  | |
| Iridaceae | *Iris pallasii* |  | |
|  | *Clinopodium chinense* | Flavonoid glycosides, Saponins, Lactones, Resin, Phenols, Organic acids | |
|  | *Clinopodium polycephalum* |  | |
|  | *Damnacanthus indicus* | Damnacanthal, Damnacanthol, Damnidin, Juzunal, Norjuzunal, Nordamnacanthal,  2-Benzylxanthopurpurin, 5- Hydroxyalizarin-*1*-methylether, Alizarin-*1*-methylether | |
| Labiatae | *Dracocephalum tanguticum* | Flavonoid glycoside, Phenols, Amino acids, Volatile oil, Steriods | |
|  | *Elsholtzia blanda* |  | |
|  | *Elsholtzia bodinier* |  | |
|  | *Isodon glaucocalyx* | Kaempferol-3-ram-glu-7-glu, Methylsulphone, Thymine | |
|  | *Isodon nervousus* |  | |
|  | *Isodon striatus* | Flavonoid glycoside, Phenols, Organic acids, Amino acids | |
|  | *Lamium barbatum* | Stachyose | |
|  | *Prunella vulgaris* | Oleanolic acid, Ursolic acid, Rutin, Hyper-oside, Caffeic acid, Delphinidin, Cyanidine,  *d*-Camphor, *d*-Fenchone | |
|  | *Salvia chinensis* | Phytosterols, Triterpene, Amino acids, Stachyose | |
|  | *Salvia miltiorrhiza* | Tanshinone I, II_A_ & II_B_, Isotanshinone I & II, Miltirone, Cryptotanshinone,  Isocrytotanshinone, Tanshinol I & II | |
|  | *Scutellaria baicalensis* | Baicalein,Baicalin,Wogonin,Wogonoside, Neo-baicalein,*β*-Sitosterol,Scutellatin, Skullcapflav-one I & II,Koganebananin,Carthamidin, Isocar-thamidin,Campsterol, 2-(3-Hydroxy-4-meth-oxyphenyl)ethyl-O-*α*-*L*-rhamnsoyl-*β*-D-(4-feruloyl)glucoside, Chrysin, Chrysin-6-*α*-*L*-ara-binoside-8-*β*-D-glucoside, Baicalin methyl ester, Wogonin-5-*β*-D-glucoside, Wognin-7-O-glucuroside methyl ester, 5,8-Dihydroxy-6,7-dimethylflavone, 2’,5,8-Trihydroxy-7-metho-xyflavone, 2’,5,8-Trihydr-oxy-6,7-dimethoxy-flavone, 4’,5,7-Trihydroxy-6-methoxyflavone, Dihydroxoxylin, Penta-hydroxyflavanone | |
|  | *Scutellaria barbata* | Alkaloids, Flavonoid glycosides, Phenols, Phytosterols | |
| Lardizabalaceae | *Akebia quinata* | Oleanolic acid- & Hederagenin-3-O-*β*-(sugar)_n_ pyranosides ( n = 1~ 5) | |
| Lauraceae | *Cassytha filiformis* | Cassyfiline, Cassythine, Cassythidine, Laurotetanine, Galactitol | |
|  | *Cinnamomum cassia* | Volatile oil, Cinnamic aldehyde, Tannins, Cinnamyl acetate, Phenylpropyl acetate,  Cinncassiols A, B, C, C_1_, C_2_, C_3_, D_1_, D_2_, D_3_ & D_4_, Cinnzeylanine, Cinnzeylanol, | |
| Lauraceae | *Cinnamomum cassia* | Anhydrocinnzeylanine, Anhydrocinnzeylanol, 19-O-*β*-Glucopyranosides of cinncassiols A, B & D_1_, Glucosides of cinncassiols C_1_, D_1_, D_2_ & D_4_, Monoacetylcinncassiol A | |
|  | *Abrus precatorius* | Abruguinone A,B,C, Orientin, Isoorientin, Abrectorin, Desmethoxycentaureidin-7-O-rutinoside, Abricin, Abridin, Precol, Abrol Abrasine, Precasine, N,N-Dimethyl-*L*-trypto-phan, Choline, Trigonelline, Precatorine, Abrine, Hypaphorine | |
|  | *Arachis hypogea* | *cis*- & *trans*-Resveratrol; Oleic, lauric, linoleic, palmitic, stearic, arachidic, behenic, cerotic, lignoceric, myristic, gadoleic, eicosenoic & hypogaic acids | |
|  | *Cassia tora* | Chrysophanol, Alaeemodin, Rhein, Rhein-glu, Emodin, Anthraquinone, Physcoin, Obtusin, Aurantioobtusin, Rubrofusarin, Torachryson, Toralactone, Vitamin A | |
| Leguminosae | *Desmodium caudatum* | Alkaloids (0.12%) | |
|  | *Desmodium pulchellum* | Alkaloids, Flavonoid, Phenols, Tannin | |
|  | *Glycyrrhiza radix* | Benzoicacid,Formononetin,Isoliguiritigenin,Liquiritigenin,4’,7-Dihydroxyflavone,Formonon-etin-7-glucoside, Liquiritin, Paeoniflorin, Isoliquiritin, Gallic & Glycyrrhizin acids | |
|  | *Glycyrrhiza uralensis* | Glycyrrhizic & glycyrrhetinic acids, Glycyrrhetic acid glucuronide, Liquiritigenin, Flavonoids, Licoricore, Licoricidin, Glycyrol, Isoglycyrol, 5-O-Methylglycyrol | |
|  | *Medicage pupulina* | Estrogen-like ingredients, Glucosides, Galactosylmannose, Proteins, Cellulose | |
|  | *Millettia speciosa* | Torachrysone, Emodin, Aloeemodin, Chryso-phanol-1-*β*-gentiobioside, Chrysophanol, Obtusin, Rhein, Aurantiobtusin, Rubrofusarin-6- *β*-gentiobioside,Toralactone, Alkaloids | |
|  | *Kummerowia striata* | Flavonoids & Glucosides, Stizolamine | |
|  | *Sophora flavescens* | *d*-Matrine,*d*-Oxymatrine, *d*-Sophoranol,5-Hyd-roxymatrine, *1*-Anagyrine, *1*-Methyl-cytisine, *1*-Baptifoline, *1*-Sophocarpine, 2-*n*-Alkyl-5,7-dihydroxy-6,8-dimethylchromone, Luteolin-7-*β*-D-glucoside, Isokurarinone, Kurarinol, Neo-kurarinol, Norkurarinol, Kuraridinol, Trifolirh-izin, (+)Matrine, (+)Isomatrine (+)Matrine-N-oxide, (+)Sophoranol, (+)-Sophoranol-N-oxide, (-)Sophocarpine, (+)-Sophocarpine-N-oxide, Sophoramine, (-)Δ^~~7~~^-Dehydrosophoramine, (-) Anagyrine, (-)N-methylcytisine, (-)Baptifoline | |
|  | *Vicia multicaulis* |  | |
| Lemnaeae | *Marsilea quadrifolia* |  | |
| Liliaceae | *Aletris spicata* |  | |
| Liliaceae | *Allium sativum* | Allicin, Alliin,Allyl sulfide, Methyl sulfide, Citral, Geraniol,Linalool, *α* & *β*-Phellandrenes, Propionic aldehyde, Pentanal, *γ*-*L*-Glut-amyl-S-methyl-*L*-cysteine & its sulfoxide,*γ*-*L*-Glutamyl -*L*-valine,*γ*-*L*-Glutamyl-*L*-leucine,*γ*-*L*-Glutamyl methionine, *γ*-*L*-Glutamyl-isoleucine, *γ*-*L*-Glut-amyl-S-allyl-*L*-cysteine,*γ*-*L*-Glutamyl-*L*-phenylalaine, *γ*-*L*-Glutamyl-S(*β*-carbosypropyl) -*L*-cysteinylglycine, Scordinin A | |
| Linaceae | *Linum usitatissimum* | Orientin,Vitexin, Linamarin; Palmitic,stearic, oleic, linoleic & linolenic acids | |
| Lindsaeaceae | *Stenoloma chasanum* | Vitexin, Protocatechualdehyde; Syringic & protocatechuic acids | |
| Lycopodiaceae | *Lycopodium cernnum* | Cernuine, Lycocernuine, Nicotine, Cernoside, *β*-Sitosterol, Stigmasterol, Campesterol, Triterpenes, Serratenediol, Serratriol, Serratriol triacetate, 16-Oxo-21-episerratriol,  21-Episerratriol, Cornoside, *α*-Onocerin | |
| Magnoliaceae | *Magnolia denudata* | Calamenene | |
|  | *Schisandra chinensis* | Wuweizisu C,Citral, Gomisin A,B,C,D, E,F,G, H,J,N,O,R, Schisandrin A,C,D,E,*γ*-Schisandrin, Deoxyschisandrin, Fumaric acid, Pregomisin, Schizandrol, Schisantherin A, C, D, (-)Gomisin L_1_, L_2_, K_1_, (+)Gomisin K_2_, K_3_, M_2_, (±)Gomisin M_1_, Angeloylgomisin H, O, P, Q,Tigloylgomi-sin H & P, Benzoylgomi-sin H, Epigomisin O, Sesquicarene,(+)- *α*-Ylangene, Chamigrenal, *α* & *β*-Chamigrene, Angeloylisogomisin O, *d*-Epigalbacine. | |
|  | *Grossypium herbaceum* | Gossypol, Flavonoids, Acetovanilone, Saponin, Phenolic carboxylic acids, Phenols, Betaine, Fatty alcohols, Sterols | |
| Malvaceae | *Malvastrum coromandelianum* |  | |
|  | *Sida mysorensis* | Ephedrine | |
| Marattiaceae | *Angiopteris officinalis* |  | |
| Melastomataceae | *Melastoma polyanthum* |  | |
| Menispermaceae | *Fibraurea tinctoria* | Palmatine, Jatrorrhizine, Columbamine, Fibranine, Fibraminine, Fibralactone | |
|  | *Stephania cepharantha* | Cepharanthine, Isotetrandrine, Cycleanine, Homoaromoline,Berbamine,Tetrandrine, Quin-ine, Papaverine, Cepharamine, Cepharanoline, Trilobine, Codeine, Liriodenine, Cepharanone A, B, Morphine, Lysicamine, Cepharadione A, B, O-Nornuciferine, Stephanine, Stesakine, Crebanine, Dehydrocrebanine | |
|  | *Cudrania cochinchinensis* | Phenols, Flavonoid glycosides, Amino acids, Organic acids, Carbohydrates | |
| Moraceae | *Cudrania tricuspidata* | Glucosides, Organic acids | |
|  | *Ficus simplicissima* |  | |
|  | *Morus alba* | Palmitic acid,Stearic acid,Betaine, *cis*- & *trans*-5-Hydroxy-*L*-pipecolic acid, Morusin, Resorci-nol, Alboctalol, Resveratrol, Oxyresveratrol, Dihydroxyresveratrol, Quercetin, Dihydrokae-mpferol, Dihydromorin, Cyclomorusin, Morin, Kuwanon A, B, C, D, E, F, G, H, I, J, Chrysan-themin, *α*- & *β*-Amyrin, Ergosterol, Mulberro-furan B, Morachalcone A, Chalcomoracin, Stigmast-5-en-3*β*-ol-7-one | |
|  | *Ardisia japonica* | Volatile oil, Bergenin, 2-Hydroxy-5-methoxy-3-pentadecenylbenzoquinone,Triterpenes, Ilexol, Quercitrin, Myricitrin, Embelin, Ardisin, Ardisinol II | |
| Myrsinaceae | *Ardisia mamillata* |  | |
|  | *Maesa indica* |  | |
| Myrtaceae | *Rhodomyrtus tomentosa* | Oleanolic, alphitolic, ursolic, betulinic & betulonic acids; 3*β*-acetoxy-11*α*,12*α*-epoxyl-eanan-28,13,*β*-dide, *α* & *β*-Amyrin,*β*-Amyrenonol, Lupeol, Betulin, Betulin-3-acetate, 21*α*-Hop-22(29)-en-3*β*,30-diol, Friedelin, Taraxerol, Campesterol, Stigmasterol | |
| Neperthaceae | *Nepenthes mirabilis* | Flavonoid glycosides, Phenols, Amino acids, Carbohydrates, Anthraquinone glycosides | |
| Oleaceae | *Jasminum officinale* | Oxalic, citric, tartaric & malic acids | |
| Orchidaceae | *Gymnadenia conopsea* | Methyl vanillin | |
|  | *Habenarie densa wall* |  | |
| Oxalidaceae | *Oxalis corniculata* | Salts of oxalate; Citric,tartaric & malic acids | |
| Pandanaceae | *Pandanus furcatus* |  | |
|  | *Pandanus tectorius* | Volatile oil, Methyl phenylethyl, Citral, Dipentene, *d*-Linalool, Phenyl ethylacetate,  Stearoptene, Ester of phthalic acid | |
| Papaveraceae | *Corydalis stricta* | Corynoline | |
|  | *Coryolalis bungeana* | Alkaloids, Lactones of coumarin, Steroid saponin, Phenols, Resin, Volatile oil | |
|  | *Meconopsis integrifolia* |  | |
|  | *Meconopsis quintuplinervia* |  | |
| Parmeliaceae | *Parmelia saxatilis* | Atranorin; Salazinic, uanetic & parmatic acids | |
|  | *Sticta pulmonacea* | Gyrophoric acid | |
| Passifforaceae | *Passiffora wilsonii* |  | |
|  | *Polygala arillata* |  | |
| Polygalaceae | *Polygala aureocauda* |  | |
|  | *Polygala caudata* | Flavonoid glycosides, Saponin, Phenols, Coumarone, Resin | |
|  | *Fagopyrum cymosum* | *p*-Coumaric & ferulic acids, Rutin, Shakuchirin, Quercetin, Quercetrin | |
|  | *Oxyria digyna* |  | |
|  | *Polygonum chinense* | Flavonoid glycosides, Shakuchirin | |
| Polygonaceae | *Polygonum cuspidatum* | Anthraquinone, Anthraquinone glycosides, Emodin, Chrysophanic acid,8-O-*β*-*D*-Glucosyl-emodin, Physcion, Physcion-8-*β*-*D*-glucoside, Resveratrol, Chrysophanol, Anthraglycosides A & B, Polydatin | |
|  | *Polygonum multiflorum* | Chrysophanic acid,Chrysophanol,Rhein, Emo-din,Chrysophanic acid,Anthrone,Rhapontin, 3,3’-Di-O-galloylprocyanidin-*β*-2-chrysophan-ol, Physcion, 2,3,5,4’-Tetrahydroxystilbene-2-O-*β*-*D*-glucopyranoside-3”-O-monogalloyl ester,Catechin, 3-O-Galloylcatechin, 3-O-Galloylepicatechin, 3-O-Galloylprocyanidin | |
|  | *Polygonum orientale* | Isovitexin,Orientin,Quercetin,3’-Hydroxy-3,4’ 5,5’,6,7,8-heptamethoxyflavone, 3,3’,4,5,5’8- Hexamethoxy-6,9-methylenedioxyflavone, 3,3’,5,6,7,8-Hexamethoxy-4’,5-methylenedi-oxyflavone | |
|  | *Rumex crispus* | Emodin, Chrysophanic acid, Chrysophanein, 1,8-Dihydroxy-3-methyl-9-anthrone | |
|  | *Rumex japonicus* | *β*-Myrcene | |
|  | *Rumex patientia* | Anthraquinones, Chrysophanol, Emodin monomethylether, Tannin | |
|  | *Adiantum flabellulatum* | Flavonoid glycosides, Phenols, Organic acids, Carbohydrates | |
| Polypodiaceae | *Cyrtomium fortunei* | Tannins, Volatile oil, Cyrtopterin, Farrerol, Astragalin, Isoquercitrin, Cyrtomin | |
|  | *Pteris multifida* | Flavonoids, Sterol, Phenols, Amino acids, Lactones | |
| Polypovaceae | *Ganoderma japonicum* | Ergosterol, Organic acids, Aminoglucose, Polysaccharide, Resin, Mannitol, Fatty acids, Alkaloids, Lactones, Coumarin, Water-soluble proteins and enzymes | |
| Primulaceae | *Lysimachia fortunei* |  | |
|  | *Lysimachia insignis* |  | |
| Pteridaceae | *Asplenium incisum* | Phenols, Aldehyde derivatives | |
| Pteridaceae | *Phymatopsis hastata* | Coumarin | |
|  | *Pteris laeta* |  | |
|  | *Pteris nervosa* |  | |
|  | *Aconitum naviculare* |  | |
|  | *Anemone rivularis* |  | |
|  | *Clematis chinensis* | Anemonin, Anemonol, Sterol, Saponin, Carbo-hydrate, Lactone, Oleanolic & amino acids, acids 3-O-*β*-1~5 sugar units, Hederagenins 3-O-*β*-1~5 sugar units, Hederagenin 23-O-*α*-*L*-arabinopyranoside, Hederagenin 23-O-*β*-*D*-glucopyranoside, Olean-12-ene-28-oic acid-3*β*,24-diol 3-O-*α*-*L*-rhamnopyranosyl- *α*-*L*-arabinopyranoside | |
|  | *Clematis finetiana* |  | |
| Ranunculaceae | *Ranunculus chinensis* |  | |
|  | *Ranunculus sceleratus* | Ranunculin, Protoanemonin, Anemonin, Pyrogallol,Tannin,Flavonoids,5-HT_1_ & 5-HT | |
|  | *Thalictrum dasycarpum* | Thalicarpine, Thalidasine | |
|  | *Thalictrum ramosum* |  | |
|  | *Berchemia giraldiana* |  | |
| Rhamnaceae | *Ziziphus jujuba Mill var inermis* | Phytosterols, Triterpins, Jujuboside A, B, Juju-bogenin, Betulin; Oleanolic, maslinic, betulic, betulonic & cumaroylmalinic acids, Ebelin lactone, Vitamin C, , Yuzirine, *trans-* & *cis*-3-O-*p*- & *trans*-2-O-*p*-Cumaroyl alphitolic acids, Yuziphine,(±)-Coclaurine, Mauritine A, Asimi-lobine, Mucronnine D, Zizybeoside I & II, Zizyvoside, Zizyvoside I, Vomifoliol, Roseo-side, 2*R* & 2*S*-6,8-Di-C-glucosylnariingenin | |
|  | *Eriobotrya japonica* | Amygdalin, Benzaldehyde, Oleanolic & ursolic acids; Loquatoside | |
|  | *Parnassia palustris* | Kaempferol, Rutin, Hyperin | |
| Rosaceae | *Rubus alceaefolius* |  | |
|  | *Rubus buergeri* |  | |
|  | *Rubus ellipticus* |  | |
|  | *Rubus parvifolius* | Tannin, Carbohydrates, Flavonoid glycosides | |
| Rhodoraceae | *Agapetes mannii* |  | |
|  | *Damnacanthus indicus* | Damnacanthal,Damnacanthol,Damnidin, Juzu-nal, Nordamnacanthal, Norjuzunal, 2-Benzyl-xanthopurpurin, Alizarin-1-methyl ether | |
|  | *Galium verum* | Palustroside, Rutin, Asperuloside, Chlorogenic acid, Methylvanillin, Piperonal, Rubiadin, Primeveroside, Pseudopurpurin glucoside | |
| Rubiaceae | *Gardenia jasminoides* | Geniposide, Gardenoside, Genipin-1-glucoside, Genipin-1-*β*-*D*-gentiobioside, Shanzhiside, Crocin, Crocetin, *D*-Mannitol, Nonacosane, *β*-Sitosterol, 10-Acetylgeniposide, Gardoside, Picrocrocinic acid, Zcandoside methyl ester, Methyl deacetylasperuloside | |
|  | *Oldenlandia chrysotricha* |  | |
|  | *Oldenlandia cantonensis* |  | |
|  | *Oldenlandia costata* | Triterpenoid saponins | |
|  | *Oldenlandia diffusa* | Hentriacontane, Stigmasterol; Ursolic, oleanolic & *p*-coumaric acids, *β*-Sitosterol, *β*-Sitosterol-*D*-glucoside | |
|  | *Paederia scandens* | Arbutin, Scandoside, Paederoside, Asperulo-side, Deacetylasperuloside, Oleanolic acid | |
|  | *Serissa Serissoides* |  | |
| Rutaceae | *Boenninghausenia albiflora* | Volatile oil, Rutin, Bergapten, Dictamnine, Matsukaze lactone, Methyl *p*-coumarate, Coumarin, 7-Methoxy-2,2-dimethylchromene, 6-(*trans*-1-Buten-3-only)-7-methoxy-coumarin, Angelical, Daphnetin-8-methylether, Isopim-pinellin, Xanthotoxin, Chalepensin, Myrcene, *α*-Phellandtene, *β*-Caryophyllene, Caryophyll-ene oxide, (-)Nodak-enetinacetate, 3-(1,1-Di-methylallyl)xanthyletin, (*E*) & (*Z*)-7-Hydroxy-6-(3-hydroxy -3-methyl-1-butenyl)-2H-1-benz-opyran-2-one, 1-Hydroxyacridone, Noracrony-cine, Rutacridone, 1-Hydroxy-N-methyl-acridone, 1,7-Dihydroxy-N-methyl-acridone | |
|  | *Clausena lansium* | Clausenamide | |
|  | *Evodia lepta* | Alkaloids | |
|  | *Phellodendron amurense* | Berberine,Magnoflorine,Phellodendrine,Candicine,Obacunonic & lumicaerubic acids; Guani-dine, Menisperine, Obaccunone, Obaculactone, Dictaminolide, Palmatine,Phellamurin,7-Dehy-drostigmasterol, *β*-Sitosterol, Campesterol, Amurensin, Phellatin, Phellavin, Hyperoside, Methyl-*n*-heptylketone, Myrcene, Limonin, Jateorrhizine | |
|  | *Zanthoxylum avicennae* | Sterols, Phenols, Organic acids, Avicine, Hesperidin, Diosin, Avicennin, Candicine, Tembetarine, Magnoglorine, Chelerythrine, | |
| Salicaceae | *Salix babylonica* | Tannins(4.93%), Iodine (0.21g/kg), Salicin(→salicylalcohol) | |
|  | *Salix matsudana* | Tannins (3.06-7.49%) | |
| Santalaceae | *Thesium longifdium* |  | |
|  | *Chrysosplenium sinicum* |  | |
| Saxifragaceae | *Philadelphus henryi* |  | |
|  | *Ribes emodense* |  | |
|  | *Saxifraga przewalskii* |  | |
| Schisandraceae | *Schisandra chinensis* | *γ*-Schizandrin, Schisantherin D, *β*-Bisabolene, *β*-Chamigrene,*α*-Ylangene,Schizandrin, *pseudo* -*γ*-Schizandrin, Deoxyschizandrin, Citral, Schi-zandrol, Gomisin A,B,C,D,E,F, G, H,J, N,O,R, Fumaric acid, Pregomisin, Angel-oylgomsin H, O, P,Q, Tigloylgomisin H & P, Epigomisin O, Benzoylgomisin H, Benzoylisogomisin O, Wu-weizisu C, Sesquicarene, Cham-igrenal, *α* & *β*-Chamigrene, (-)Gomisin K_1_,L_1_, & L_2_, (+)Gomisin K_2_ & K_3_, (±)Gomisin M_1_, M_2_ | |
| Schizaeaceae | *Lygodium japonicum* | Lygodin | |
|  | *Brandisia hancei* |  | |
|  | *Lindernia crustacea* |  | |
|  | *Melasma arvense* |  | |
|  | *Pedicularis rex* |  | |
| Scrophulariac-eae | *Rehmannia glutinosa* | *β*-Sitosterol, Stigmasterol, Phytosterols, Camp-esterol, Rehmannin, Catalpol, Alkaloids, *γ-*Amino butyric & amino acids, Glucosamine, Phosphoric & fatty acids,, Sugars , Luteolin, Chrysoeriol, Rehmannioside A, B, C, D, Dihydrocatalpol, Monomelittoside, Leonuride, Aucubin, Melittoside | |
|  | *Siphonostegia chinensis* | Cardiac glycosides and volatile oil | |
|  | *Striga masuria* |  | |
|  | *Veronica ciliata* |  | |
|  | *Veronicastrum axillare* | Sterol, Mannitol, Tannin, Resin, Carbohydrates, Inorganic salts | |
| Selaginellaceae | *Selaginella doedderleinii* | Alkaloids, Reducing substances, Phytosterols & saponins | |
|  | *Selaginella moellendorcii* | Phenols and two aldehyde derivatives | |
| Solanaceae | *Lycium barbarum L.* | Vitamin B_1_, B_2_, C, *β*-Sitosterol, Linoleic acid | |
|  | *Lycium chinense* | Betaine, Rutin, Vitamin C, *β*-Sitosterol-*β*-*D*-glucoside, Inosine, Hypoxanthin; Cytidylic, uridylic & amino acids | |
| Solanaceae | *Solanum lyratum* | Tomatidenol, Solasodine, *α*-Soladulcidine, Soladulcidine, Solasonine, Solamargine, Yamogenin, Tomatida-3,5-diene, *α*-,*β*- & *γ*-Soladulcine, 15*α*-Hydroxysoladulcidine,15*α*-Hydroxysolasodine, 15*α*-Hydroxytomatid-ine,15α-Hydroxytomatidenol, Saponin(hydro-lysis →3-O-(*β*-*D*-gluco-pyranosyl(1→2)-*β*-*D*-glucopyranos-yl(1→4)-*β*-*D*-galacopyranosyl spirostanol derivatives & mixture of tigogenin, neotigogenin, diosgenin and yamogenin | |
|  | *Solanum nigrum* | SNL glycoprotein | |
| Sparganiaceae | *Sparganium stoloniferum* | Volatile oil (0.05%) | |
| Taccaceae | *Tacca plantaginea* |  | |
|  | *Annesla fragranas* |  | |
| Theaceae | *Camellia sinensis* | Caffeine,Theophylline, Theobromine, Xanthine Galloyl-*l*-epigallocatechol,*l*-Epicatechol, Volat-ile oil, *β,γ*-Heptenol, *α,β*-Heptenal, *α*-& *β*-Iono-ne, *α*-Terpineol, 2,4-Decadienal, 3,7-Dimethyl-1,5,7-octatrien-3-ol-2-phenyl-2-butenal, Theaspirone, Jasmone, *δ*-Pilgerol, Furfuryl alcohol, *α*-Muurolene,Benzyl formate, Theafolisaponin, Indole, Phenylethyl formate, Geranial, Pyrrole-2-alde-hyde, 2-Hexenyl benzoate, Vitamin C, Methyl-phenyl carbinol, Carotene, Dihydroergosterol, Flavonoids, Theasapogenol A,B, C, E, Quercetin-*D*-fructosyl-*β*-*D*-glucoside, Naringenin, (+)Gallo-catechin, 5-Hydroxycampenoside, Boschniak-ine, Camelliagenin A & D, Campenoside, | |
| Thymelaceae | *Daphne genkwa* | Genkwanin, Hydroxygenkwanin, Apigenin, Genkwadaphnin, Daphnetoxin,Yuanhuafin,  Yuanhuadin,Yuanhuacin, Benzoic acid, | |
|  | *Angelica sinensis* | Ferulic acid sodium | |
|  | *Bupleurum chinense* | Saikosaponin A, C, D | |
| Umbelliferae | *Centella asiatica* | Isothankunic, betulic, brahmic & madasiatic acids, Asiaticoside, Thankuniside, Isothankuni-side,Mesoinositol,Madecassoside, Drahmoside, Brahminoside, Centellose, Carotenoids, | |
|  | *Hydrocotyl sibthorpioides* | Coumarin, Hyperin, Hyperoside, Quercetin, Phenols, Amino acid, Volatile oil | |
| Urticaceae | *Pellionia repens* |  | |
|  | *Pilea plataniflora* |  | |
| Valerianaceae | *Patrinia rupestris* | Volatile oil, Saponin (hydrolized to produce oleanolic acid), Alkaloids | |
|  | *Callicarpa nudiflora* |  | |
| Verbenaceae | *Clerodendron cyrtophyllum* | Clerodendrin A (a diterpenoid) | |
|  | *Verbena officinalis* | Tannins, Volatile oil, Verbenalin, *β*-Cantene, Cardiotonic glycoside, Hastatoside | |
|  | *Ampelopis brevipedunculata* | Tannins, Sterols, Triterpenoid, Cardiac glycoside | |
|  |  |  |  |
| Vitaceae | *Tetrastigma hemsleyanum* |  | |
|  | *Vitis thunbergii* |  | |
| Zingiberaceae | *Curcuma aromatica* | Camphene, *α*-Camphene, *α*-Camphor, *l*-*α*-Curcumene, *α*-& *β*-Curcumene, Curcumin, Demethoxycurcumin, Bisdemethoxycurcumin, Carvone,Turmerone, Arturmerone  *p*-Tolylmethylcarbinoldiferuloylmethane | |
|  | *Curcuma longa* | Curcumin, Demethoxycurcumin,Turmerone, Carvone, *p*-Tolylmethylcarbinoldiferuloyl-methane, *d*-*α*-Phellandrene, Dihydroturmerone | |

***** Jiangsu New Medical College: *Traditional Chinese Medicine Dictionary (Vols 1, 2, 3)*. Shanghai: Shanghai Science and Technology Publising House; 1979.

**a.** a**_1_** Norbaek R, *et al. Photochem* 2002; 60(4):357-9; Kisiel W, Zielinska K. *Photochem* 2001; 57(4):523-7.

a**_2_** Matthaus B, Ozcan M. *J Agricultural & Food Chemistry* 2005; 53(18):7136-41.

a**_3_** Ali A, *et al.* *Journal of Asian Natural Products Research* 2003; 5(2):137-42; Upadhyay RK, *et al.* *Journal of Asian Natural Products Research* 2001;3(3):207-12.

Pawar RS, Bhutani KK. *Phytomedicine* 2005; 12(5):391-3.

a**_4_** Taylor & Francis. *International Journal of Toxicology* 2001; 20 Suppl 2: 79-84.

a**_5_** Lim KT, *et al*. *Journal of Medicinal Food* 2005; 8(2):215-6; 8(1):69-77; 2004; 7(3): 349-57.
