# Supplementary material for: A survey of Chinese herbal ingredients with liver protection activities
Source: Chin Med. 2007 May 10;2:5. doi: 10.1186/1749-8546-2-5 (PMC1876451; doi:10.1186/1749-8546-2-5)
Supplement: Additional File 2 — Representative structures of compounds with potential hepatoprotection and their key physicochemical parameters (MW, Clog P, CMR, μ and Elumo). The table presents the chemical structures of the compounds with potential liver protection and the corresponding physicochemical parameters, namely MW, Clog P, CMR, μ and Elumo. [file 1749-8546-2-5-S2.doc]

**Representative structures of compounds with potential hepatoprotection and their key physicochemical parameters (MW, Clog P, CMR, μ and E_lumo_)**

| ***** Herbs | Compounds | Structures | Comments | Refs | |
| --- | --- | --- | --- | --- | --- |
|  |  |  |  |  | |
| *Schisandra chinensis*  (*Wuweizi*) | Deoxyschisandrin (Schisandrin A)  MW = 416.52  Clog P=5.23  CMR=11.47  μ = 2.81; E_lumo_= 0.17 |  | A neolignan with 6 methoxyl  groups | 10 | |
| *Clausena lansium*  (*Huangpi*) | Clausenamide  MW = 297.36  Clog P=1.89; CMR=8.52  μ = 2.61; E_lumo_= 0.26 |  | Optically active cyclic amide | 11 | |
| *Corydalis*  *stricta*  (*Zijin*) | Corynoline  MW = 367.41  Clog P=2.14; CMR=9.72  μ = 1.63; E_lumo_= -0.10 |  | A tetrahydro-iso-quinoline derivative | 12 | |
| *Kopsia officinalis*  (*Yunnan Ruimu*) | Kopsinine  MW = 338.45  Clog P=2.38; CMR=9.52  μ = 0.52; E_lumo_= 0.50 |  | An aspido-spermane type alkaloid | 13 | |
|  |  |  |  |  |  |
| *Artemisia capillaris*  (*Yinchen*) | Capillarisin  MW = 300.27  Clog P=2.40; CMR=7.90  μ = 3.36; E_lumo_= -0.64 |  | A flavonoid with one methoxy and three phenolic OH groups | 14 | |
| *Silybum marianum*  (*Shuifeiji*） | Silybin  MW =482.45  ClogP=1.94;CMR=12.02  μ = 1.92; E_lumo_= -0.83 |  | with 3 phenolic OH group | 15 | |
| *Bupleurum chinense*  (*Chaihu*) | Saikosaponin A  MW =781.00  ClogP=4.34;CMR=19.87  μ = 3.03; E_lumo_= 1.07 |  | A saponin (nonionic surfactant) may improve microcirculation | 16 | |
| *Akebia quinata* (Thunb.) Decne  (*Bayuezha*） | Oleanolic acid 3-O-β- (sugar)_n_ pyranoside;n=2~5  ClogP = 1.24 ~ 6.72  CMR = 18.70 ~ 28.57  MW =721.0~1191.4  E_lumo_=1.11~1.28  μ = 1.77~7.58 |  | A saponin with 2 ~ 5 sugar residues | 17, 19 |  |
|  | Hederagenin 3-O-β- (sugar)_n_ pyranoside;n=1~5  ClogP = -0.05~ 6.4  CMR = 16.10 ~ 28.72  MW =604.8 ~1207.4  E_lumo_=1.13~1.23  μ = 1.30~6.12 |  | A saponin with 2 ~ 5 sugar residues | 17, 18, 19 |  |
|  | Melittoside(R=O-Glc)  MW=524.5 Clogp= -6.0 CMR=11.37 E_lumo_= 0.42  μ = 4.60  Monomelittoside(R=OH)  MW = 362.3  ClogP= -5.06 CMR=8.00  E_lumo_= 0.61 μ = 4.46  Aucubin (R=H)MW=346.3  Clogp=-4.03 CMR=7.85  E_lumo_= 0.65 μ = 4.43 |  | A glucoside with 2 hydroxyl group | 20 |  |
| Rehmannia glutinosa  (*Gandihuang*) | Leonuride(R= H)  MW = 348.4 CMR=7.87 ClogP=-3.19 E_lumo_= 1.20  μ = 2.27  Rehmannioside C;R=Gal  MW = 510.5 CMR=10.78  ClogP=-4.63 E_lumo_= 1.16 μ = 2.05 |  | Glucoside with 1 or 2 hydroxyl groups | 20 |  |
|  | Rehmannioside B  MW =538.6;CMR=11.14  ClogP=-5.30 E_lumo_= 0.73 μ = 7.06 |  | Glucosides with 1 hydroxyl and 1 epoxy groups | 20 |  |
|  | Luteolin(R=H);MW=286.2  ClogP = 2.31;CMR = 7.29  E_lumo_= -1.02 μ = 4.64  Chrysoeriol (R=Me)  ClogP = 2.75C; MR = 7.75  E_lumo_=-0.96 μ = 4.53 |  | A flavonoid with 4(or 3) phenolic OH groups | 21 |  |
|  | Methyl p-coumarate  MW = 208.22  ClogP = 1.95  CMR = 5.16  E_lumo_= -0.72 μ = 1.87 |  | A phenolic group and *β*-unsaturated acid ester | 22, 23 |  |
|  | Coumarin  MW =146.15  Clogp=1.41 CMR=4.16  E_lumo_= -0.93 μ = 4.82 |  |  | 24 |  |
|  | Osthole  MW = 244.29  ClogP=3.74 CMR=7.07  E_lumo_= -0.91 μ = 3.77 |  |  | 23-27 |  |
| *Boenning- hausenia*  *Albiflora*  (*Yanjiaocao*) | 1-Hydroxyacridone  R=R_2_=R_1_=H;MW=211.2  ClogP=2.51 CMR=6.22  E_lumo_= -0.36 μ = 4.33  1-OH-N-Me-acridone  R=R_2_=H; R_1_=Me MW=225.3  ClogP=2.88 CMR=6.68  E_lumo_= -0.34 μ = 4.60  1,7DiOH-N-MeAcridone  R=OH; R_1_=Me R_2_=H  MW = 241.3  ClogP=2.33 CMR=6.84  E_lumo_= -0.42 μ = 5.71 | Rutacridone(R=H; R_1_=Me R_2_=) MW=307.4  ClogP=4.16 CMR=8.95  E_lumo_= -0.31 μ = 5.37  Noracronycine(R=H, R_1_=Me; R2= )  MW = 307.4  ClogP=4.34 CMR=8.95  E_lumo_= -0.41 μ = 5.16 |  | 28, 29 |  |
|  | Dictamine  MW =199.21  ClogP=3.01  CMR =5.68  E_lumo_= -0.73; μ = 3.74 |  |  | 30 |  |
|  | Rutin  MW = 610.5  Clog P = -2.50  CMR = 13.56  E_lumo_= -1.03; μ = 5.98 |  | A flavonoid with 4 phenolic OH groups | 15 |  |
| *Boenning- hausenia*  *Albiflora*  (*Yanjiaocao*) | Caryophyllene Oxide  MW =220.36  ClogP=4.74;CMR =6.46  E_lumo_= 1.10; μ = 2.11 |  |  | 31 |  |
|  | *α*-Phellandrene  MW =136.24  ClogP = 4.41 CMR=4.59  E_lumo_= 0.50; μ = 0.24 |  |  | 31 |  |
|  | *β*- Caryophylene  MW = 204.36  ClogP=6.45;CMR= 6.54  E_lumo_= 1.16; μ = 0.49 |  |  | 31 |  |
| *Magnolia denudata*  Desr.  (*Xinyi*) | Calamenene  MW =202.34  ClogP= 5.74;CMR= 6.70  E_lumo_= 0.55; μ = 0.27 |  |  | 32 |  |
| *Rumex japonicus* Houtt.  (*Yangti*) | *β*-Myrcene  MW = 136.24  ClogP=4.33; CMR=4.89  E_lumo_= 0.51; μ = 0.28 |  |  | 33 |  |
| Glycyrrhiza *uralensis*  (*Gancao*) | Glycyrrhizin  MW =822.98  ClogP=1.89 CMR=19.96  E_lumo_= 0.03; μ = 7.01 |  |  | 34 |  |
|  | 18*β*-Glycyrrhetinic acid  MW = 470.7  ClogP=6.29 CMR=13.30  E_lumo_= 0.05; μ = 5.83 |  |  | 34 |  |

***** Jiangsu New Medical College: *Traditional Chinese Medicine Dictionary (Vols 1, 2, 3)*. Shanghai: Shanghai Science and Technology Publising House; 1979.
