# Supplementary material for: A survey of Chinese herbal ingredients with liver protection activities
Source: Chin Med. 2007 May 10;2:5. doi: 10.1186/1749-8546-2-5 (PMC1876451; doi:10.1186/1749-8546-2-5)
Supplement: Additional File 3 — The hepatoprotective compounds and the calculated physicochemical parameters used in analysis. The data provided represent the physicochemical properties of the compounds with potential hepatoprotection. [file 1749-8546-2-5-S3.doc]

**The hepatoprotective compounds and the calculated physicochemical parameters used in analysis**

| Compound names | ^a^MW | ^b^ClogP | ^b^CMR | ^c^μ | ^c^E_homo_ | ^c^E_lumo_ | ^c^H_f_ |
| --- | --- | --- | --- | --- | --- | --- | --- |
|  | **(**Dalton) (Debyes) (eV ) (eV) (Kcal/mol) | | | | | | |
| **Oleanolic Acids** |  |  |  |  |  |  |  |
| Oleanolic acid 3-O-*α*-*L*-rha-*α*-*L*-arapyranoside | 735.0 | 6.72 | 19.16 | 2.02 | -9.22 | 1.26 | -558.3 |
| Oleanolic acid 3-O-*β*-xyl-*α*-*L*-arabinopyranoside | 721.0 | 6.20 | 18.70 | 4.82 | -9.24 | 1.20 | -560.3 |
| Olean-12-ene-23-oic acid 3*β*, 24-diol(4-epihederagenin)3-O-α-*L*-rha-α-L-arabino-pyranoside | 751.0 | 5.43 | 19.13 | 1.77 | -9.27 | 1.21 | -605.8 |
| Oleanolic acid 3-O-*β*-*D*-xyl-*α*-*L*-rha-*α*-*L*-arabino-pyranoside | 867.1 | 5.89 | 21.91 | 1.93 | -9.20 | 1.28 | -741.3 |
| Oleanolic acid 3-O-*β*-*D*-rib-*α*-*L*-rha-*α*-*L*-arabino-pyranoside | 867.1 | 5.89 | 21.91 | 3.01 | -9.19 | 1.27 | -742.9 |
| Saikosaponin A | 781.0 | 4.34 | 19.87 | 3.03 | -9.57 | 1.07 | -622.5 |
| Oleanolic acid 3-O-*β*-ribo-α-*L*-rha-*α*-arapyranoside | 1029.0 | 4.36 | 25.29 | 2.87 | -9.20 | 1.21 | -974.4 |
| Oleanolic acid 3-O- *β*-*D*-glu-β-*D*-xylo-*α*-*L*-rha-*α*-*L*-arabinopyranoside | 1029.2 | 3.43 | 25.20 | 3.86 | -9.22 | 1.25 | -970.2 |
| Oleanolic acid 3-O-*β*-*D-L*-glu-*β*-*D*-glu-*β*-*D*-ribo-*α*- rha-*α*-arabinopyranoside | 1191.4 | 1.24 | 28.57 | 6.68 | -9.17 | 1.11 | -1204.3 |
| Oleanolic acid 3-O-*β*-glu-*β*-*D*-glu-*β*-*D*-xylo-*α*-*L*-rha-*α*-arabinopyranoside | 1191.4 | 1.24 | 28.57 | 7.58 | -9.13 | 1.16 | -1202.5 |
| Hederagenins | | | | | | |  |
| Hederagenin 3-O-*α*-*L*-arabinopyranoside | 604.8 | 6.40 | 16.10 | 2.96 | -9.19 | 1.23 | -416.7 |
| Hederagenin 23-O-*α* –*L*-arabinopyranoside | 604.8 | 6.40 | 16.10 | 3.84 | -9.29 | 1.18 | -420.4 |
| Hederagenin 23-O- *β*-*D*-glucopyranoside | 634.9 | 6.37 | 16.71 | 3.71 | -9.35 | 1.13 | -468.8 |
| Hederagenin-3-O-*α*-*L*-rha -*α* -*L*-arabinopyranoside | 751.0 | 5.43 | 19.31 | 5.81 | -9.20 | 1.13 | -1248.0 |
| Hederagenin 3-O-*β*-*D*-xylopyranosyl-*α*-*L*-rha-*α*-*L*-arabinopyranoside | 883.1 | 4.59 | 22.02 | 1.30 | -9.23 | 1.22 | -783.8 |
| Hederagenin3-O-*β*-*D*-rubo -rha-*α*-arabinopyranoside | 883.1 | 4.59 | 22.07 | 1.79 | -9.23 | 1.23 | -784.6 |
| Hederagenin 3-O-*β*-*D*-glu-  *β*-*D*-ribo-rha-*α*-*L*-arabino-pyranoside | 1045.2 | 3.06 | 25.44 | 1.89 | -9.26 | 1.22 | -1017.4 |

| Hederagenin 3-O-*β*-*D*-glu-*β*-*D*-xylo-*α*-*L*-rha-*α*-*L*-arabinopyranoside | 1045.2 | 2.13 | 25.35 | 2.91 | -9.31 | 1.18 | -1018.2 |
| --- | --- | --- | --- | --- | --- | --- | --- |
| Hederagenin-3-O-*β*-*D*-glu -*β*-*D*-glu-*β*-*D*-ribo-*α*-L-rha -*α*-*L*-arabino-pyranoside | 1207.4 | -0.05 | 28.72 | 5.81 | -9.20 | 1.13 | -1248.0 |
| Hederagenin3-O-*β*-glu-*β*-*D*-glu-*β*-*D*-xylo-*α*-*L*-rha-*α*-*L*-abinopyranoside | 1207.4 | -0.05 | 28.72 | 6.12 | -9.16 | 1.19 | -1246.2 |
| Glycyrrizin | 823.0 | 1.89 | 19.96 | 7.01 | -9.88 | 0.03 | -755.1 |
| 18*β*-Glycyrrhetinic acid | 470.7 | 6.29 | 13.30 | 5.83 | -9.87 | 0.05 | -218.9 |
| Polysaccharides | | | | | |  |  |
| Manninotriose | 488.5 | -5.37 | 10.44 | 2.29 | -10.13 | 2.12 | -766.4 |
| Raffinose | 504.5 | -4.66 | 10.44 | 4.22 | -10.27 | 1.75 | -759.1 |
| Stachyose | 666.6 | -6.24 | 13.81 | 4.75 | -10.05 | 1.78 | -1220.2 |
| Verbascose | 828.7 | -7.82 | 17.18 | 5.37 | -10.30 | 1.64 | -1225.4 |
| Rehmanniosides |  |  |  |  |  |  |  |
| Catalpol | 362.3 | -4.38 | 7.76 | 3.01 | -9.70 | 0.73 | -409.9 |
| Rehmannioside B | 538.6 | -5.30 | 11.14 | 7.06 | -9.68 | 0.73 | -633.0 |
| Rehmannioside C | 510.5 | -4.63 | 10.78 | 2.05 | -9.35 | 1.16 | -646.3 |
| Melittoside | 524.5 | -5.98 | 11.37 | 4.60 | -9.79 | 0.42 | -662.7 |
| Monomelittoside | 362.3 | -5.06 | 8.00 | 4.46 | -9.76 | 0.61 | -433.2 |
| Leonuride | 348.4 | -3.19 | 7.87 | 2.27 | -9.30 | 1.20 | -414.6 |
| Aucubin | 346.3 | -4.03 | 7.85 | 4.43 | -9.52 | 0.65 | -390.6 |
| Xanthine Derivatives |  | | | |  |  |  |
| Caffeine | 194.2 | -0.06 | 4.99 | 3.66 | -8.94 | -0.32 | 9.1 |
| Theophylline | 180.2 | -0.06 | 4.53 | 3.30 | -9.07 | -0.37 | 4.7 |
| Alkenes & Alkanes |  | | | | |  |  |
| Myrcene | 136.2 | 4.33 | 4.89 | 0.28 | -9.22 | 0.51 | 18.6 |
| Clausenamide | 297.4 | 1.89 | 8.52 | 2.61 | -9.40 | 0.26 | -70.6 |
| Kopsinine | 338.5 | 2.38 | 9.52 | 0.52 | -8.54 | 0.50 | -42.3 |
| Corynoline | 367.4 | 2.14 | 9.72 | 1.63 | -8.79 | -0.10 | -103.3 |
| *α*-Phellandrene | 136.2 | 4.41 | 4.59 | 0.24 | -8.65 | 0.50 | -4.7 |
| *β*-Caryophyllene | 204.4 | 6.45 | 6.54 | 0.49 | -9.25 | 1.16 | 2.0 |
| Caryophyllene oxide | 220.4 | 4.74 | 6.46 | 2.11 | -9.72 | 1.10 | -14.2 |
| Calamenene | 202.3 | 5.74 | 6.70 | 0.27 | -8.93 | 0.55 | -20.7 |
| Dictamine | 199.2 | 3.01 | 5.68 | 3.74 | -8.60 | -0.73 | 19.1 |
| Rutacridone | 307.4 | 4.61 | 8.95 | 5.26 | -8.45 | -0.32 | -27.1 |
| Noracronycine | 307.4 | 4.34 | 8.95 | 5.06 | -8.39 | -0.40 | -30.4 |
| 1-Hydroxyacridone | 211.2 | 2.51 | 6.22 | 4.33 | -8.46 | -0.36 | -18.21 |
| 1-HO-N-methylacridone | 225.3 | 2.88 | 6.68 | 4.60 | -8.37 | -0.34 | -7.69 |
| 1,7-Dihydroxy-N-methylacridone | 241.3 | 2.33 | 6.84 | 5.71 | -8.21 | -0.42 | -50.10 |

| Camelliagenins & Theasapogenols | | |  |  |  |  |  |
| --- | --- | --- | --- | --- | --- | --- | --- |
| Camelliagenin A | 458.7 | 6.47 | 13.46 | 2.96 | -8.96 | 1.48 | -233.0 |
| Camelliagenin C | 458.7 | 5.14 | 13.61 | 1.93 | -9.37 | 1.03 | -284.0 |
| Camelliagenin D | 474.7 | 5.24 | 13.61 | 3.38 | -9.05 | 1.37 | -275.0 |
| Theasapogenol B | 474.7 | 5.24 | 13.61 | 1.62 | -9.35 | 1.06 | -282.3 |
| Theasapogenol A | 490.7 | 3.90 | 13.77 | 2.85 | -9.32 | 1.10 | -325.1 |
| Theasapogenol E | 488.7 | 3.88 | 13.65 | 4.89 | -9.50 | 0.66 | -313.4 |
| Campenosides & Flavonoids | | |  |  |  |  |  |
| Campenoside | 490.5 | 0.74 | 12.51 | 1.95 | -9.41 | -0.71 | -395.5 |
| 5-Hydroxy-campenoside | 506.5 | -0.19 | 12.66 | 1.45 | -9.40 | -0.73 | -436.3 |
| Rutin | 610.5 | -2.50 | 13.56 | 5.98 | -8.86 | -1.03 | -626.2 |
| Silybin | 482.5 | 1.94 | 12.02 | 1.92 | -9.12 | -0.83 | -319.6 |
| Luteolin | 286.2 | 2.31 | 7.29 | 4.64 | -9.08 | -1.02 | -173.0 |
| Naringenin | 272.3 | 2.43 | 7.06 | 1.74 | -9.23 | -0.57 | -153.0 |
| Chrysoeriol | 300.3 | 2.75 | 7.75 | 4.53 | -9.00 | -0.96 | -164.4 |
| Capillarisin | 300.3 | 2.40 | 7.90 | 3.36 | -9.23 | -0.64 | -192.6 |
| Aromatic Compounds | | | |  |  |  |  |
| Boschniakine | 161.2 | 1.14 | 4.77 | 1.54 | -9.89 | -0.65 | -13.6 |
| Methyl *p*-coumarate | 208.2 | 1.95 | 5.16 | 1.87 | -9.05 | -0.72 | -92.9 |
| Coumarin | 146.2 | 1.41 | 4.16 | 4.82 | -9.46 | -0.93 | -30.5 |
| Osthole | 244.3 | 3.74 | 7.07 | 3.77 | -9.21 | -0.91 | -68.6 |
| Angelical | 204.2 | 1.16 | 5.28 | 1.94 | -9.44 | -1.07 | -99.9 |
| 6-(trans-1-Buten-3-onyl) -methoxycourmarin | 244.3 | 1.81 | 6.95 | 2.52 | -9.08 | -1.04 | -91.9 |
| (*E*)7-hydroxy-6-(3-hydr- oxy-3-methyl-1-butenyl) -2H-1-benzopyran-2-one | 246.3 | 1.79 | 6.84 | 4.78 | -8.83 | -0.88 | -118.1 |
| (*Z*)Isomer(stated above) | 246.3 | 1.79 | 6.84 | 3.96 | -9.29 | -1.05 | -114.5 |
| Daphentin 8-methylether | 192.2 | 1.33 | 4.93 | 3.35 | -9.19 | -0.98 | -111.1 |
| Xanthotoxin | 216.2 | 2.30 | 5.68 | 4.54 | -8.85 | -1.00 | -63.7 |
| Bergapten | 216.2 | 2.30 | 5.68 | 6.28 | -9.12 | -1.02 | -66.03 |
| Isopimpinellin | 246.2 | 2.33 | 6.30 | 5.63 | -8.98 | -1.07 | -98.7 |
| Deoxyschisandrin | 416.5 | 5.23 | 11.47 | 2.81 | -8.56 | 0.17 | -181.3 |
| (+)-Gallocatechin | 290.3 | 1.13 | 7.18 | 1.97 | -9.13 | -0.13 | -211.8 |
| Matsukaze lactone | 350.3 | 2.11 | 9.38 | 8.18 | -8.90 | -1.12 | -123.4 |
| 7-Methoxy-2,2-dimethyl -chromene | 190.2 | 3.63 | 5.57 | 1.22 | -8.39 | -0.03 | -40.9 |
| (-)-Nodakenetin acetate | 258.3 | 3.27 | 8.04 | 9.26 | -8.96 | -0.74 | -146.8 |
| Chalepensin | 254.3 | 3.99 | 7.65 | 5.23 | -9.07 | -0.96 | -21.7 |
| 3-(1,1-Dimethylallyl)- xanthyletin | 296.4 | 5.09 | 8.73 | 5.83 | -8.71 | -0.78 | -45.87 |

**a.** calculated from the structures by ISIS Draw 2.3 **b.** calculated by using ClogP version 4.0; **c.** cal-culated from HyperChem 5.0 program after performing geometry optimization and energy minimization using AM1 semiempirical method, and using the Polak-Riviere conjugate gradient with RHF spin pairing, 0.01 convergence limit in vacuum and RMS gradient of kcal/(Åmol). **c.** Descripters for SAR analysis because these parameters, especially Clog P and E_lumo_, are most often used to correlate with biological activities [57-60]. Examination of these parameters reveals some similarities and differences among the subgroups of these compounds.
